# Process oriented guided inquiry learning (POGIL®) marginally effects student achievement measures but substantially increases the odds of passing a course

**DOI:** 10.1371/journal.pone.0186203

**Published:** 2017-10-12

**Authors:** Lindsey Walker, Abdi-Rizak M. Warfa

**Affiliations:** Department of Biology Teaching and Learning, University of Minnesota, Minneapolis, Minnesota, United States of America; Georgetown University Medical Center, UNITED STATES

## Abstract

While the inquiry approach to science teaching has been widely recommended as an epistemic mechanism to promote deep content understanding, there is also increased expectation that process and other transferable skills should be integral part of science pedagogy. To test the hypothesis that coupling process skills to content teaching impacts academic success measures, we meta-analyzed twenty-one studies (*n* = 21) involving 7876 students that compared Process Oriented Guided Inquiry Learning (POGIL), a pedagogy that provides opportunities for improving process skills during content learning through guided-inquiry activities, to standard lecture conditions. Based on conventional measures of class performance, POGIL had a small effect on achievement outcomes (effect size = 0.29, [95% CI = 0.15–0.43]) but substantially improved the odds of passing a class (odds ratio = 2.02, [95% CI: 1.45–2.83]). That is, participants in the POGIL pedagogy had higher odds of passing a course and roughly performed 0.3 standard deviations higher on achievement measures than participants in standard lectures. In relative risk terms, POGIL reduced the risk of failing a course by 38%. These findings suggest providing opportunities to improve process skills during class instruction does not inhibit content learning but enhances conventional success measures. We compare these findings with those of recent large meta-analysis that examined the effects of global active learning methods on achievement outcomes and course failure rates in science, technology, engineering, and mathematics (STEM) fields.

## Introduction

There is increased expectation that science education should encompass practices that embody the ways of doing science and the epistemic practices of the scientific enterprise [1–5]. By science practices, we mean the acts of posing questions, generating and testing hypothesis, analyzing and interpreting data, engaging in arguments from evidence, as well as transferrable process skills of collaboration, team leadership, and participating communal activities [5,6]. To this end, there have been various research-based instructional practices that have been widely adopted since the mid-1980s to improve science process skills [3,5]. Process Oriented Guided Inquiry Learning (POGIL), with roots in chemistry but now widely used across range of disciplines, is one such pedagogy that provides opportunities for students to develop and improve specific process skills during science content learning [7–10].

In the work reported here, we focus on POGIL to answer the question, “how does coupling teaching scientific practices to content learning impact student success measures?” POGIL provides an appropriate venue to address this question as POGIL materials use a learning cycle based on three phases of inquiry: exploration of a model, concept invention, and application [7]. This provides opportunities for students to engage in process skills that go above and beyond content and emphasize the process of integrating knowledge [11]. Moog [7] specifically describes seven process skills that can be developed in a POGIL learning environment when using a well-designed POGIL activity: communication, teamwork, management, information processing, critical thinking, problem solving, and assessment (specifically self-assessment). That is, POGIL materials are designed to develop transferable skills in the context of content learning, with one or two process skill targets in well-designed POGIL activities [7,12]. To realize these, students in a POGIL classroom take up individual roles within their small group learning communities (i.e., manager, reporter, skeptic) to establish positive interdependence [7,8]. We therefore contend that POGIL provides an environment in which students encounter scientific practices and processes as normal part of their classroom activities. Thus, synthesis of empirical studies that examine the effectiveness of POGIL can indirectly shed light on how coupling process skills to content learning impacts student performance measures.

We had a second motive for conducting this study. In their recent large meta-analysis, Freeman and colleagues [13] examined the global effect of active learning variants on student performance measures. As such, the authors intentionally used an “all-inclusive” definition of what counts as active learning, from the occasional use of worksheets during lecture to activities in reformed classrooms with higher student engagement, a point criticized by some as too broad to be meaningful [14,15]. By focusing on a well-defined active learning method, POGIL, we were interested to understand whether a birds’ eye view of a clear active-learning pedagogy will confirm or refute the findings of the Freeman meta-analysis. We thus asked three specific questions in this study:

1. What is the impact of POGIL pedagogy on student achievement outcomes compared to standard lecture?
2. What are the odds of passing a course using POGIL instruction as opposed to standard lectures?
3. Do the findings of this study with respect to achievement and course pass/failure rates falsify or verify earlier meta-analysis that examined the global effects of active learning on these variables?

Our working null hypothesis were that 1) *H*_*01*_: POGIL pedagogy is no more effective than standard lectures in improving student achievement outcomes, and 2) *H*_*02*_: the odds of passing a POGIL classroom are no better than passing in a standard lecture classroom. Here, we focus on pass rates as measure of student success and retention as opposed to the commonly reported failure rates in the literature [8,12]. Course failure rates measure the percentage of students who receive grades of D, F or withdraw from a course for any reason. Pass rates, on the other hand, measure the percentage of students who successfully complete a course with at least a grade of C or better and therefore persist to further course work. As many STEM courses require a grade of C or better in pre-requisite courses, pass rates may be better indicators of student success and persistence than simply measuring failure rates [16] and thus are the variable of interest in this study.

**Fig 1:**
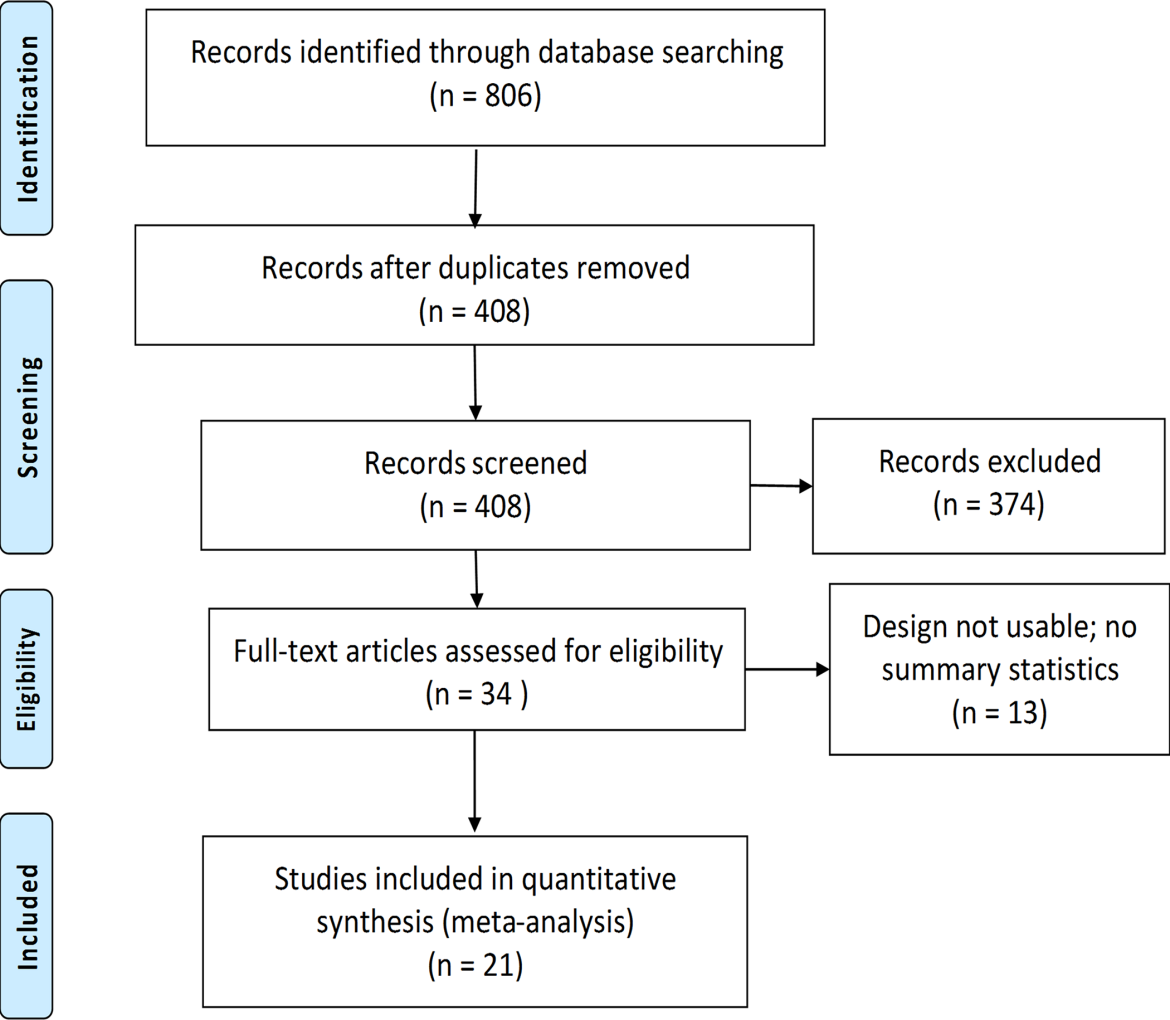
PRISMA flow diagram showing publication selection process. Publication selection involved success phases of de-duplication, surface level screening, study design check, and study admission/inclusion analyses.

To test our proposed hypotheses, we conducted an exhaustive search of empirical studies on POGIL pedagogy, a search that yielded an initial list of over 800 relevant studies which, through successive phases of screening and exclusion [Fig 1, see Methods section for full details], led to a finale sample of 21 primary studies [9, 10, 17–35]. We meta-analyzed these studies to address our research questions, generating 35 effect sizes that contrasted POGIL pedagogy with lecture as a standard control group.

## Methods

Following the recommendations of the PRISMA Statement [36] for systematic review and meta-analysis reporting, S1 Text provides a checklist of all items included in our meta-analysis. The PRISMA Statement consists of 27-item checklist [36] most of which we report. The following subsections provides full details of all the procedures we followed and our analyses methods.

### Literature search

Using previously established protocols [13, 37], we broadly searched literature on POGIL in four online databases (Web of Science, Scopus, ERIC and Google scholar), mined reviews and bibliographies and used snowballing strategy of admitted studies [13] to further locate potential publications. For each search, we used the following query terms: POGIL or Process-Oriented Guided-Inquiry Learning (with or without the dash). Since initial query on Google scholar returned over 270,000 hits, we refined it by adding “control group” or “treatment group” as specific filters of this search engine. Using previously described procedure [37], we randomly analyzed a sample of the Google scholar hits to ensure the additional filters did not result in loss of relevant work. This initial search yielded over 800 POGIL-related publications for possible consideration [Fig 1].

### Selection and inclusion

In successive phases of selection [Fig 1], we screened publications for potential inclusion, based on the following inclusion criteria: (*i*) study reported POGIL intervention in the context of naturalistic setting; (*ii*) participants were identified as high school or college/university students; (*iii*) study reported quantitative measures of student achievement with sufficient statistical information to enable analysis (e.g., mean, standard deviation, F output, etc.) and/or course pass/failure numbers for all participants; and (*iv*) the study design was experimental or quasi-experimental (control group and pre-posttest design).

De-duplication of the initial hits resulted 408 studies for further consideration [Fig 1]. This was followed by surface level screening, based on article titles and abstract or consultation of full articles when study characteristics were not evident, that further winnowed down potential materials to 34 studies. Using previously established protocols [13,37], both authors subsequently reviewed and coded all 34 articles independently and reached consensus on whether a study met the admission criteria and contained the data we needed to compute effect sizes [13]. This final step of selection and inclusion resulted 21 studies [Fig 1] that we subsequently meta-analyzed (all the analyzed articles are found in S1 Table and indicated with asterisks in the reference list). We note that we could not tell from the analyzed articles whether all the studies in the experimental group strictly composed of POGIL activities or the frequency with which they enacted POGIL activities. The criteria we used relied on author-reported identification of their study as a POGIL intervention (criterion *i*) and the presence of empirical data that compared the POGIL intervention with standard lectures (criterion *iii*). The final set of the selected articles included studies conducted both at college (n = 19) and high school (n = 2) settings. Most of the studies reported content coverage as being similar between the POGIL classrooms and the comparison standard lectures (i.e., content coverage and study settings remained identical apart from the introduction of POGIL as intervention). Inter-rater agreement between the authors on study selection and inclusion, as measured by Cohen’s kappa, was strong [κ = 0.817, z = 4.85, *p* < 0.001].

### Moderator variables

To facilitate data selection and analysis, we organized the data into a spreadsheet on Excel (S2 Text). We noted in the spreadsheet if the study took place in a high school or college setting, in a chemistry or non-chemistry discipline, class size, and teacher training on POGIL use (S2 Text). By teacher training, we mean if the study reported whether an instructor participated introductory POGIL workshops or another training on how to use POGIL materials and facilitate POGIL classrooms. The POGIL project offers frequent one and three-day trainings available to those who show interest in this pedagogy. Disciplinary subfields, class size, and instructor training were the only moderators on effect size that we could extract from the selected studies.

We used a previously established protocol [37] to designate a class as small if the number of treatment subjects was 50 or less, medium if the number was between 51 and 100, or large if there were more than hundred participants. Because POGIL pedagogy was initially used in chemistry departments [7–9], most studies (*n* = 15) were from chemistry and there was a scattering of POGIL use in other STEM disciplines in the articles identified for inclusion. We thus used “chemistry” and “other” as possible “discipline” moderator variables on effect sizes. We did not use study setting (high school or college) as a variable because of the limited number of studies done at the high school level (n = 2) making it unsuitable for meta-analysis purposes. In addition to meta-analyses by the individual variables, we similarly used meta-regression to assess their effects on summary outcome measures.

### Data analysis

We did all statistical computing and analyses in the R package metafor [38, 39]. We used previously established protocol [37] to compute achievement effect sizes and course pass/failure odds ratio as described by Freeman *et al*. [13]. Because individual studies might have used concept inventories, standardized exams, or instructor-constructed exams [13], the conversion of these divergent success measures to a common metric (*i*.*e*., an effect size) made plausible quantitative comparison of these studies [37]. That is, we used information present in each study to express relevant outcomes to a common scale as we and others previously described [13, 37, 40]. Briefly, we used Hedge’s [41] weighted standardized mean difference (*g*) to compute individual effect sizes of the respective studies and their variances for the achievement outcomes and the log-odds ratio for course pass/failure rates [13]. Since sample characteristics, student populations and POGIL use is likely to vary across studies, we used random-effects model [37, 38] to compute effect sizes. For ease of interpretation, the log-odds ratio for pass/failure data were converted to odds ratio and relative risks [13, 38].

When a study reported multiple outcomes for both achievement outcomes and pass/failure rate data, we aggregated the individual effect sizes into a single summary *g*-value if the reported outcomes were statistically equivalent [37]. For example, if a study reported multiple outcomes for the same subjects, such as midterm and final exams, our approach was to aggregate those multiple outcomes into a summary effect size for such study. On the other hand, if a study reported multiple outcomes but used different subsamples, then we considered such outcomes to be statistically independent and retained the individual effect sizes. For example, Straumanis and Simons [10] reported multi-institutional assessment of the use of POGIL in organic chemistry. While their data came from four different institutions (A–D), the authors sometimes reported multiple outcomes for individual institutions. We considered data involving the different institutions as independent and therefore computed separate effect sizes for each institution. However, when Straumanis and Simons reported multiple outcomes for individual institutions, we considered those to be statistically equivalent and computed summary effect size for the individual institutions. As described previously [37], this procedure allowed us to resolve statistical dependencies of the analyzed studies and to avoid inflating the computed summary effect sizes.

We used Q-statistical analyses (Q_E_ and Q_M_) to detect variability in effect size outcomes [42]. The statistic Q_E_ measures the amount of heterogeneity in an estimated effect size and, when significant, suggests moderator influence [37, 42]. Q_M_ measures the amount of heterogeneity accounted for by moderator variables. A statistically significant Q_M_ suggests the moderator of interest has significant effect on the measured outcome [37, 38, 42]. We thus computed both Q_E_ and Q_M_ to measure variability in the estimated effect size outcomes.

As described by Borenstein *et al*. [43], we evaluated publication bias visually at outcome-level by examining funnel plot symmetry (S2 Fig) and statistically by using Egger’s regression test and rank correlation test in the metafor package [39]. We augmented the publication bias analyses by calculating fail-safe N values using the Rosenthal approach [44] and the more conservative Orwin approach [45].

## Results

### Achievement measures

**Fig 2:**
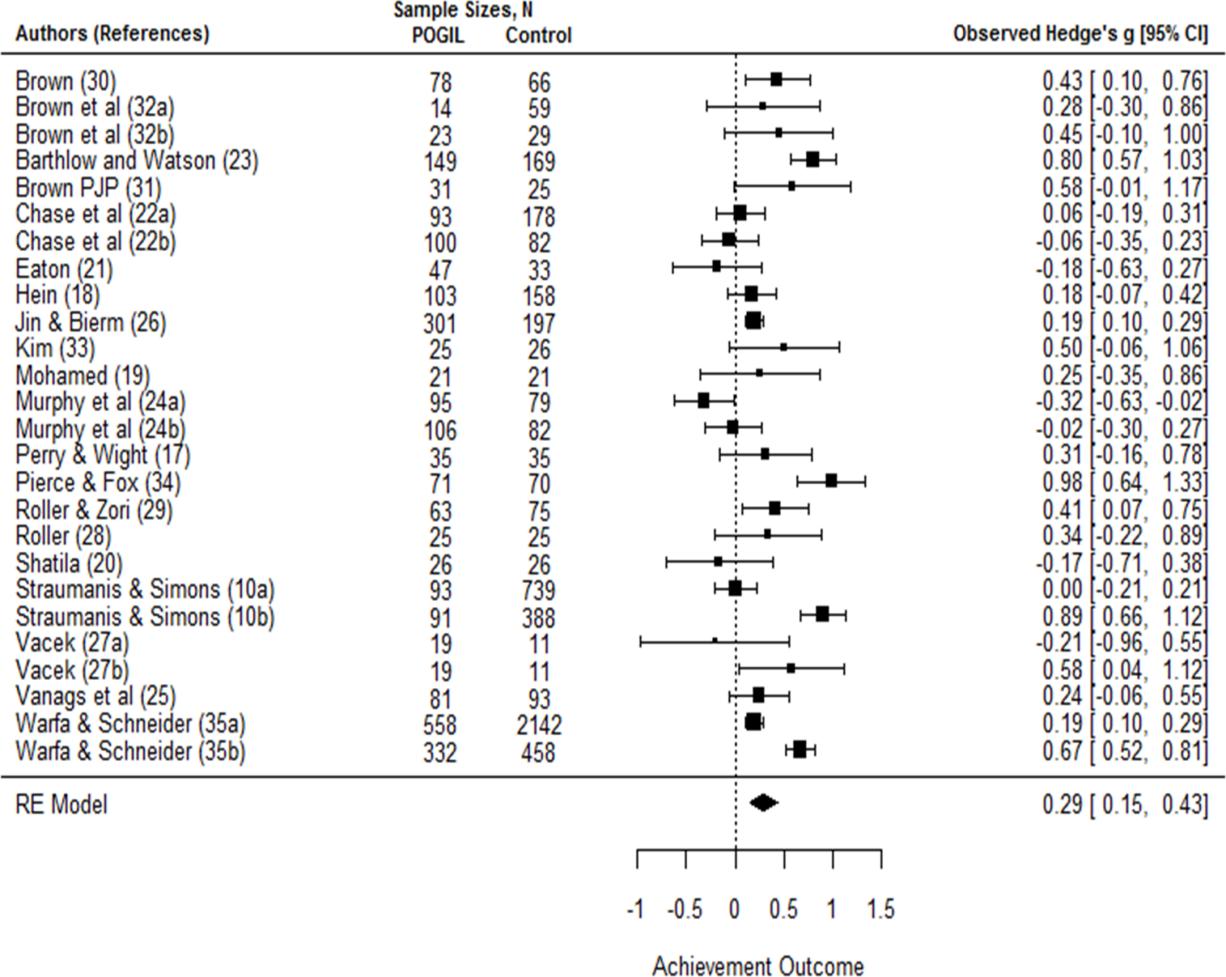
Effect sizes for achievement. The forest plot shows the results of twenty-six experiments from 20 studies that contrasted POGIL pedagogy with control groups. The summary effect size of 0.29 [95% CI = 0.15–0.43] suggests student performance in the POGIL treatment is 0.3 standard deviations higher than that of the control group.

Based on weighted standardized mean difference of independent studies (*n* = 20) that contrasted POGIL pedagogy with traditional lecturing (*N*_*pogil*_ = 2599, *N*_*control*_ = 5277, *N*_*total*_ = 7876), POGIL had a small but statistically significant effect on student achievement measures with an overall positive effect size [*g*_*+*_ = 0.29, 95% CI = 0.15 to 0.43, Fig 2]. The summary effect size of 0.29 suggests the average achievement outcome in the POGIL cohort is roughly 0.3 standard deviations higher than that of the control group. In practical terms, this suggests a median student performance in summative assessment measures (i.e., exams or concept inventories) in a POGIL group would be at the 62^nd^ percentile compared to that of a student in a standard lecture group performing at the 50^th^ percentile. The null hypothesis of POGIL having no effect on achievement outcomes can therefore be rejected [z = 4.12, *p* < 0.0001]. Rather, POGIL appears to improve student achievement outcomes.

Heterogeneity test of the estimated achievement effect indicated considerable heterogeneity among the true effects [Q = 143.17, *df* = 25, *p* < 0.0001], suggesting significant variations in the analyzed studies. This finding is not surprising since differences in methods and study characteristics likely introduce variability among the true effects. For example, the effectiveness of POGIL may depend on class size, with participants in smaller classes benefitting from greater student-teacher interactions [13, 37]. It is also possible that instructor training may impact implementation practices [22] or how POGIL is used across the academic disciplines may vary.

To test these hypotheses, we meta-regressed the achievement omnibus effect by class size and disciplinary domains [Table 1], the only variables reported in the meta-analyzed studies with sufficient information to make the analysis meaningful. For purposes of domain analysis (see Method’s section), we combined all disciplines other than chemistry as “other” since there were few studies in the other sub-disciplines we analyzed. Thus, we had two domains with sufficient information for meta-analysis purposes: chemistry (*n* = 15) and other disciplines (*n* = 11). Both variables, class size and disciplinary domain, were not significant moderators [*Q*_*M*_ = 2.22, *df* = 3, *p* = 0.527]. However, the test for residual heterogeneity was significant [*Q*_*E*_ = 142.90, *df* = 22, *p* < 0.0001], possibly indicating other moderators not considered in our model are influencing the summary effect size. For example, it is possible that variations in how the different disciplines assess student learning influence observed effect sizes. It is also likely student perceptions of content difficulty and whether innovations in instructional strategies help them is a factor. Methodologically, most of these studies lack fidelity of implementation measures, which could explain most of the heterogeneity in the data. These hypotheses are speculations on our part–all we can say is that there are unaccounted moderators in our models that may explain the observed variations.

**Table 1:** Meta-regression of achievement outcome by moderating variables.

Variable	B	SE^a^	95% Confidence Intervals		Heterogeneity	
			Lower Level	Upper Level	*Q*_*M*_ *(df)*	*Q*_*E*_ *(df)*
*Achievement Variables (n)*						
Intercept	0.299*^b^	0.137	0.031	0.567	2.22 (3)	142.90 (22)***
Small Classes (≤ 50)	-0.160	0.193	-0.538	0.217		
Medium Classes (51–100)	-0.104	0.181	-0.458	0.251		
Non-Chemistry Disciplines	0.220	0.155	-0.084	0.523		

^a^Abbreviations: SE = standard error, *df* = degrees of freedom; n = class sizes. Effect sizes for achievement were computed based on weighted standardized mean differences (Hedge’s *g*).

^b^Stars represent conventional significance levels as follows

***p < 0.001.

**p < 0.01.

*p < 0.05.

+*p* < 0.1.

**Fig 3:**
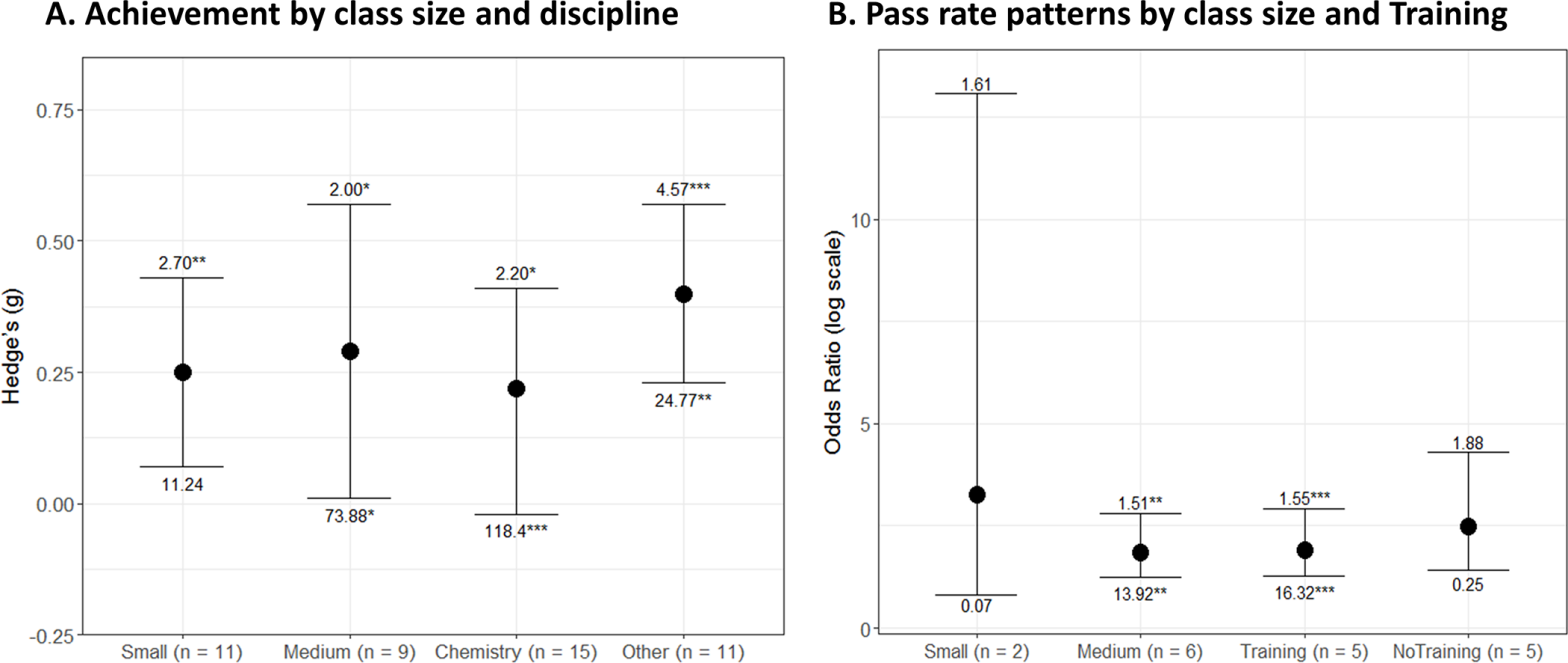
Meta-analysis by class size, disciplinary domains and instructor training. Panels A and B show random-effects meta-analytic summaries [g or odds ratio plus 95% confidence intervals (CI)] for achievement outcomes (panel A) and course pass rates (panel B). Along with conventional significance levels (***p < 0.001, **p < 0.01, *p < 0.05, +*p* < 0.1), the numbers above the error bars show effect estimates divided by their standard errors (*effect*/SE) and the numbers below the error bars show the amount of heterogeneity in each dataset (Q). *N* refers to the number of studies meta-analyzed under each condition. See main text for degrees of freedom and further details of each factor.

To further assess the influences of class size and disciplinary domains on achievement measures, we meta-analyzed the studies by these factors. Classes with 50 students or less (small) and those with 51–100 students (medium) showed significant positive small effects favoring POGIL over standard lectures [*g*_*small*_ = 0.25, 95% CI = 0.069–0.430, *p* = 0.007, *n* = 11; *g*_*medium*_ = 0.29, 95% CI = 0.006–0.574, *p* = 0.045, *n* = 9; Fig 3A]. We didn’t meta-analyze large classes as only six experiments met this criterion in our study (*n* > 100 students) and thus not enough experiments for meta-analysis purposes. Heterogeneity was nonsignificant in the small classroom data [Q = 11.23, *df* = 10, *p* = 0.339] but significant in the medium classroom data [Q = 73.88, *df* = 8, *p* = 0.045], suggesting underlying variations in study structures in medium classes contributed to substantial heterogeneity in this dataset. Synthesis across these studies therefore suggests a “general” small positive POGIL effect on achievement outcomes in small and medium classroom settings compared to standard lectures.

The summary effect size for achievement from chemistry studies [*n* = 15 studies, *g* = 0.22, 95% CI = - 0.024–0.414, Fig 3A] was significant (z = 2.20, *p* = 0.028) and roughly 2 times lower than that of other disciplines (*n* = 11) who reported significant medium effect size favoring POGIL over standard lectures [*g* = 0.40, 95% CI = 0.229–0.574, *p* < 0.0001, Fig 3A]. This finding is consistent with previous studies that report effect sizes in chemistry tend to be lower than those of other STEM subdisciplines. For example, Ruiz-Primo and colleagues [46] examined the effects of course innovations in STEM courses and reported an effect size of 0.27 for chemistry versus 0.54 for biology and 0.59 for physics. Bowen [40] similarly examined the effects of cooperative learning on chemistry and reported an effect size of 0.37. The lower effect sizes in chemistry observed in this and the previous studies, and across diverse active learning variants, may be due to the nature and culture of assessment in this subfield. We note that there was significant heterogeneity in both chemistry and non-chemistry datasets [Q_*chemistry*_ = 118.40 *df* = 14, *p* < 0.0001; Q_*other*_ = 24.77, *df* = 10, *p* = 0.006]. This analysis indicates that POGIL pedagogy was more effective than the control group to varying degrees in both chemistry and the other domains analyzed.

### Course pass rates

**Fig 4:**
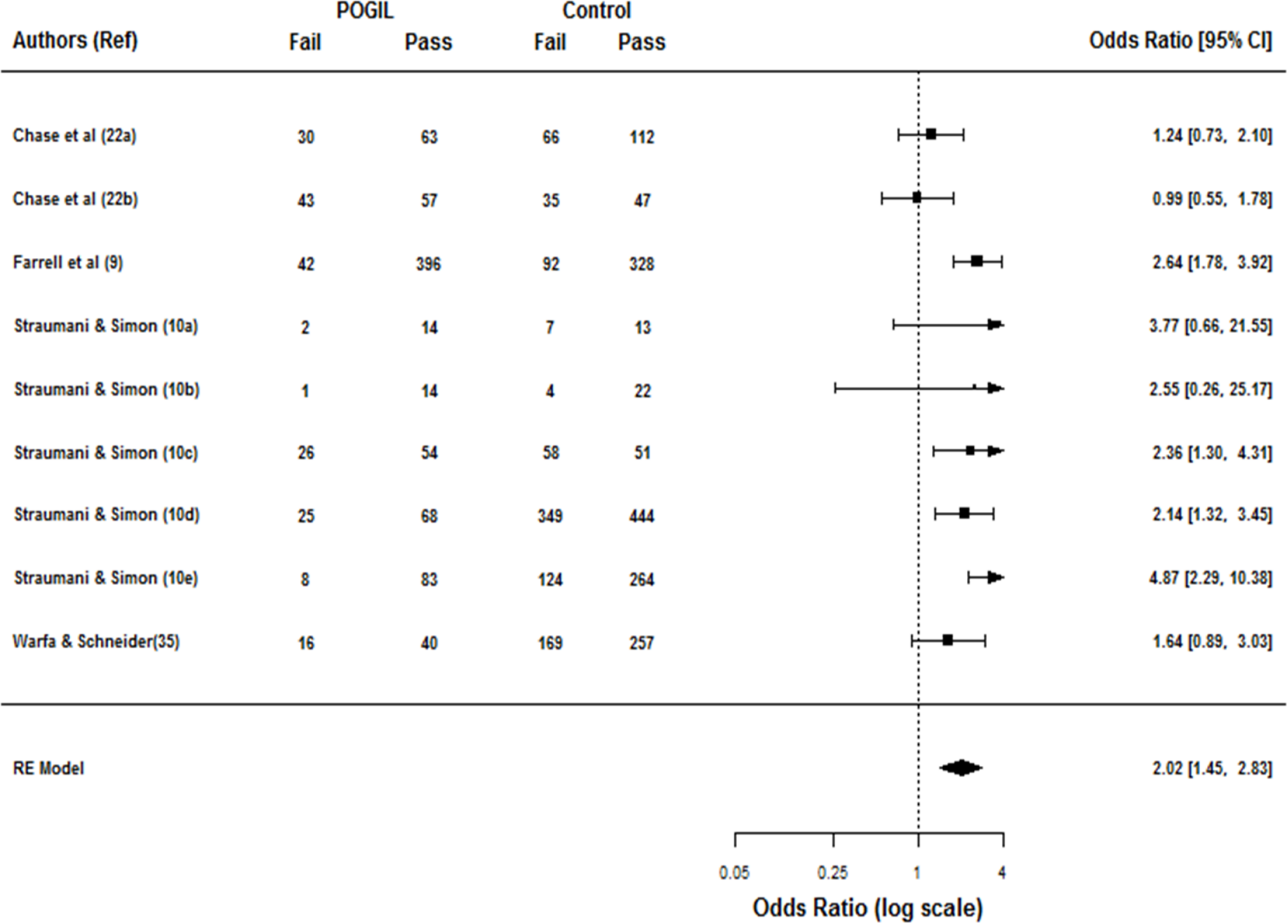
Forest plot of nine studies examining the impact of POGIL on course pass rates. The number of students receiving grades of D, F, or W (Fail) versus C or better (Pass) in each study is shown. The summary odds ratio of 2.02 [95% CI = 1.45–2.83] suggests the odds of passing were approximately two times higher in a POGIL classroom as opposed to odds of passing when taught by standard lecture.

To test the null hypothesis that the odds of passing a classroom using POGIL instruction are no better than those of a class using standard lectures, we computed the odds ratio for passing a POGIL course versus control (*n* = 9). Our analysis found an overall estimated odds ratio of 2.02 [95% CI: 1.45–2.83, Fig 4]. This suggests that the odds of passing were two times higher in a POGIL classroom versus a standard lecture classroom. In terms of relative risks, we found POGIL pedagogy reduces the risk of failing by almost 38% [relative risk of failure = 0.62, 95% CI: 0.47–0.80, S1 Fig]. The null hypothesis of the odds of passing a POGIL classroom being no better than those in a standard lecture classroom can therefore be rejected (*z* = 4.113, *p* < 0.0001). However, the test for heterogeneity [Q = 17.22, *df* = 8, *p* = 0.0279] suggested considerable heterogeneity among the true effects that warranted further analysis.

To examine the influence of moderators on the odds ratio, we meta-regressed course pass rates by class size and instructor training, the only variables with sufficient information reported in the meta-analyzed studies [Table 2]. While the results of these variables did not influence the odds ratio for passing (*Q*_*M*_ = 1.01, *df* = 3, *p* = 0.799), the test for residual heterogeneity was significant (*Q*_*E*_ = 13.044, *df* = 5, *p* = 0.023), possibly indicating that other moderators not considered in the model are influencing the pass rates.

**Table 2:** Meta-regression of course pass rates by moderating variables.

Variable	B	SE^a^	95% Confidence Intervals		Heterogeneity	
			Lower Level	Upper Level	*Q*_*M*_ *(df)*	*Q*_*E*_ *(df)*
*Pass Rate Variables (n)*						
Intercept	3.511	2.183	0.761	16.226	1.01 (3)	13.04 (5)**^b^
Small Classes (50)	0.921	3.019	0.106	8.030		
Medium Classes (51–100)	0.672	1.742	0.227	1.994		
Training	0.753	1.824	0.232	2.445		

^a^Abbreviations: SE = standard error, *df* = degrees of freedom; n = class sizes. Pass rate effect sizes are from odds ratio analysis. There were not enough studies to meta-analyze class sizes with more than 100 student enrollees.

^b^Stars represent conventional significance levels as follows

***p < 0.001.

**p < 0.01.

*p < 0.05.

+*p* < 0.1.

We further meta-analyzed pass rate patterns by class size and instructor training. Classes with 51–100 students (medium) showed significant odds ratio favoring POGIL over standard lectures [odds ratio_*medium*_ = 1.86, 95% CI = 1.23–2.80, *p* = 0.003, *n* = 6; Fig 3B]. Classes with 50 students or less (small) similarly showed statistically small odds ratio favoring POGIL over traditional lectures [odds ratio_*small*_ 3.26 = 95% CI = 0.82–13.07, *p* = 0.095] but this interpretation is suspect due to small number of studies in this category [*n* = 2]. Heterogeneity was statistically significant in the medium classroom data [Q = 13.92, *df* = 5, *p* = 0.016], suggesting underlying variations in study structures in medium classes contributed to substantial heterogeneity in this dataset. Synthesis across these studies suggests “general” odds ratio pattern favoring POGIL over standard lectures.

The odds for passing a course were roughly two times higher in a POGIL classroom versus standard lectures regardless of whether the studies reported instructor training [*n* = 6 studies, odds ratio = 1.92, 95% CI = 1.27–2.91, *p* = 0.0021, Fig 3B] or no such training [*n* = 3, odds ratio = 2.49, 95% CI = 1.43–4.31, *p* = 0.0012, Fig 3B]. There was significant heterogeneity in the studies reporting instructor training [Q_*traininy*_ = 16.32, *df* = 15, *p* = 0.006] versus those that did not report such training [Q_*no training*_ = 0.25, *df* = 2, *p* = 0.884]. The general trend of these studies therefore suggests the odds of passing a class favor POGIL pedagogy over standard lectures.

### Confirmatory findings

The recent Freeman meta-analysis (13) examined the global effect of active learning strategies on STEM disciplines. One criticism leveled against this meta-analysis was the authors’ use of an all-encompassing definition of active learning. Given that our study focused on specific, well-defined active learning strategy, we sought to refute or confirm the findings of the Freeman meta-analysis. Table 3 provides side-by-side comparison of key findings from the present study and the Freeman meta-analysis. Except for the mean achievement effect size (*g* of 0.47 in the Freeman meta-analysis vs. 0.29 in the present study), all other findings were almost identical. The average failure rates under active learning in the Freeman meta-analysis was 21.8% vs. 22.3% under the POGIL conditions in this study. The percent failure rates in traditional classrooms under the Freeman meta-analysis was 33.8% vs. 35.65% in this study. That is, standard lectures increased failure rates by 55% in the Freeman meta-analysis vs. 59.8% under the present study. The odds ratio favoring active learning over standard lectures in the Freeman meta-analysis (odds ratio = 1.95) is practically indistinguishable from the odds ratio obtained in the present study (odds ratio = 2.02). Similarly, the relative risk terms for failing were practically indistinguishable in both studies (relative risk of 0.64 vs. 0.62). Therefore, our results confirm the major findings of the Freeman meta-analysis even though ours was more narrower and focused on specific active learning strategy. We suspect similar outcomes are likely if individual active learning methods are analyzed in isolation in similar manner.

**Table 3:** Comparison of major findings from present study versus Freeman meta-analysis.

Variable	Freeman meta-analysis (Ref. 13)	Present study
Achievement outcome effect size	0.47	0.29
Percent of students who failed active learning class	21.8%	22.31%
Percent of students who failed traditional lecture class	33.8%	35.65%
Percent increase in failure rates under traditional lecturing	55%	59.8%
The odds ratio favoring active learning vs. traditional	1.95	2.02
Average relative risk for failing	0.64	0.62

### Sensitivity and diagnostic analyses

We used funnel plot and regression analyses [43] to examine whether distribution outcomes in our sample suggested publication bias (*i*.*e*., whether studies with a statistically significant effect, which are more likely to be published than those with null results, skewed the summary effect). The funnel plot, generated by plotting study outcomes (effect sizes) as a function of study precision (standard errors in the effect sizes), is a useful visualization of publication bias. Based on funnel plot inspection (S2 Fig), we found no evidence of publication bias. Furthermore, all regression tests were statistically nonsignificant [Egger’s regression test, z = – 0.323, *p* = 0.745; rank correlation test, Kendall’s tau = 0.118, *p* = 0.402], suggesting lack of asymmetry in the funnel plot for achievement outcomes. The results were similar for course pass/failure rate analysis [Egger’s regression test, z = 0.695, *p* = 0.487; rank correlation test, Kendall’s tau = 0.056, *p* = 0.920].

In addition to the funnel plot analyses, we computed fail-safe *N* analyses [43] to determine how many studies with effect size of zero should be added to the data in order estimated effect sizes to become trivial [37]. Using the Rosenthal approach [43, 44], the fail-safe numbers were quite high: 991 for achievement outcomes and 132 for course pass rates. Using the more conservative Orwin approach [43, 45], the number of studies analyzed would need to double (fail-safe *N* of 26 for achievement outcomes and *N* of 9 for course pass rates) for the outcomes to become nonsignificant.

## Discussion

There is a renewed interest in how science is taught, with national calls that science pedagogy should encompass the development of content knowledge as well as process and transferable skills [12]. Part of this drive stems from a widely reported gap between science graduate’s technical content knowledge and employer-desired transferable skill sets [12, 47–49]. However, despite the reported gap, teaching process skills often takes a back seat to developing content knowledge–in part because of the fear that developing process skills will come at the cost of content coverage [50]. In this report, we used process oriented guided inquiry learning (POGIL) to demonstrate how opportunities to develop process skills in the context of content learning impacts conventional measures of student performance and success. Our results suggest providing opportunities to develop process skills during class instruction does not inhibit content learning and leads to measurable student success rates in course achievement outcomes and pass/failure rates.

When we meta-analyzed independent studies that contrasted the use of POGIL instruction with standard lectures using equivalent exams and concept inventories, we found POGIL improved student achievement outcomes by 0.3 standard deviations. In practical terms, this suggests 62% of the POGIL cohort were above the mean of the control group and that there is 58% chance a student picked at random from the POGIL group will have a higher score than a student picked at random from the cohort taught by standard lectures. This result is comparable to the findings of earlier meta-analyses that showed various active learning strategies improved student achievement outcomes in STEM disciplines by 0.3–0.51 standard deviations [37, 40, 46, 51]. While the previous meta-analyses mainly looked at the effect of active-learning on achievement outcomes, our focus on POGIL provides indirect empirical evidence on the value of developing process skills during content instruction.

An important finding in our study relates to course pass/failure rates as measure of student retention, a phenomenon that remains problematic in STEM disciplines where substantial number of students who initially indicate interest in these fields switch out [13, 52]. One way to turn the tide around and retain more students in the sciences appears to be increasing course pass rates and the level of student engagement [53]. Here, we found that the odds of passing a POGIL class were approximately two times that of the odds of passing a class using standard lectures [odds ratio = 2.02]. In terms of relative risk, POGIL reduced the risk of failing a course by 38% while standard lectures increased the risk of failure by 59% (failure rates of 22.31% under POGIL vs. 35.65% under standard lectures). These findings are consistent with the results of a recent meta-analysis [13] that reported active learning strategies in STEM reduce failure rates by 36% (relative risk of 0.64) while traditional lectures increase failure rates by 55%. The reproducibility and the robustness of this finding has clear implications for science pedagogy in the STEM disciplines.

While the studies we examined did not directly compare proficiency gains in process skills in the courses using POGIL versus standard lecture, the design of POGIL materials emphasize developing process skills along with content learning, making POGIL a good tool to assess the common refrain that developing soft skills comes at the cost of content coverage [9, 11, 12]. While some agonize about engaging students in activities that take away time from covering content, there is a greater value in helping students learn how to use scientific practices to solve societal problems. Wright [50] specifically argues that content mastery “naturally emerges as students seek out, evaluate, and organize the information they need to develop an informed understanding about an issue.” Our analysis of the POGIL instruction certainly suggests engaging students in activities that focus on process skills does not inhibit content mastery as illustrated by the higher achievement effect sizes obtained by the students in the POGIL cohort versus those who received standard lectures.

Providing opportunities to develop and improve process skills along with content may also explain why students in the POGIL cohort had two times higher odds to pass a course than those who received standard lectures. By engaging in process skills that leverage scientific practices, it is possible the POGIL students leveraged those skill sets to solve content-specific problems. This conclusion is consistent with those of an earlier study that found lack of certain science process skills (e.g., the ability to interpret graphs or analyze data) was an important determinant of student success or failure in introductory biology course [54].

### Study limitations

The evidence we reported here suggests that POGIL, and thus the development of process skills during class instruction, has positive effect on both student achievement outcomes and course pass failure rates. However, there was, as one would expect in meta-analysis synthesis, considerable heterogeneity and variation across the studies we analyzed. While this is an inherit limitation of most meta-analyses, our use of random effects models (see Methods section) takes into consideration natural variations expected when studies are fundamentally different. That is, if the meta-analyzed studies were all identical and there was no measurable variation in them, we would have used the more appropriate fixed-effects model in that case [37, 55]. Thus, for the purposes of our synthesis, understanding how developing process skills effects content learning and course success rates, variation across the analyzed studies was necessary outcome as many of these studies involved different student samples.

A more problematic limitation of our study arises from what is reported in the primary studies we meta-analyzed. To understand what accounts for observed variations in meta-analysis results, it is often essential to investigate possible moderator effects–for example, in the context of POGIL, teacher training. Unfortunately, most studies did not report variables that would have enabled us to examine their effect on the omnibus effect sizes. The only variables reported in the studies were class size, teacher training, and discipline. When these variables were not sufficient to explain observed heterogeneity, this left us to conclude that “other moderators” not considered in our models were accounting for the observed variations across the studies.

The greatest threat to the present findings arise from the limited number of meta-analyzed studies (*n* = 21). While there was large number of studies that reported POGIL use, we excluded most from consideration because they did not report summary statistics that would have allowed us to calculate effect sizes or percentages or raw numbers to tabulate course pass/failure rates. Ruiz-Primo and colleagues [46] have previously noted the inadequate reporting of descriptive statistics in studies reporting impact of undergraduate science course innovations on student learning. This still appears to be a problem in most of the published studies. Like Ruiz-Primo and colleagues, we similarly excluded many studies because of poor quality design that did not meet our inclusion criteria (i.e., studies must use experimental or quasi-experimental design).

Publication bias could be an issue in most meta-analytic studies, especially those analyzing small number of publications. However, in this study, fail-safe *N* analyses and funnel plot diagnostics did not suggest publication bias. Given that we could only locate 21 studies that met our stringent criteria for inclusion, it is unlikely we missed the over 100 theoretical studies that would be required to make the effect sizes we computed statistically nonsignificant. Based on this body of evidence, practitioners can have overall confidence in the findings of the present study.

Another apparent limitation of the analyzed studies involves the lack of information about the nature and the frequency of training instructors received before using POGIL materials in their classrooms. Unfortunately, despite the importance of fidelity of implementation measures on the effectiveness of evidence-based instructional pedagogies, most studies do not measure or report critical elements that may influence their impact [22]. For example, for the POGIL studies we analyzed here, there was not sufficient information about how frequent the POGIL practitioners were trained to use POGIL or their level of experience with the POGIL materials (novices or long-time users?). These are all modulators that would have an effect yet were not reported.

Finally, one important variable that was not included in our analysis is if the POGIL classrooms were using learning materials that have been endorsed by The POGIL Project as true “POGIL activities.” The Project currently has an endorsement process [56] to evaluate activities for their “POGILness” although we suspect this was not the case in early stages of the Project when most of the articles we analyzed were published. Thus, it is likely some of the studies we analyzed used activities that were not endorsed by the POGIL project. While this is a limitation worth noting, we do not believe it diminishes the overall finding of the study as most of these studies have been peer-reviewed and published as POGIL studies.

## Supporting information

S1 Text PRISMA 2009 checklist on items included in the meta-analysis. (DOC)

S2 Text Spreadsheet showing organization of moderator variables. (DOCX)

S1 Table Individual and aggregate effect size of all meta-analyzed studies. For studies that reported multiple outcomes, their individual effect sizes were aggregated into one summary effect size when we found the outcomes to be statistically equivalent.
(DOCX)

S1 Fig Relative risk of course failure. The relative risk of failing a course taught by the POGIL approach versus one taught by standard lecture is depicted as a forest plot [RR = 0.62, 95% CI: 0.47–0.80]. The POGIL approach reduced the relative risk of failure by 38%.
(TIF)

S2 Fig Funnel plot assessment of publication bias. The lack of symmetry suggests the absence of publication bias even when there is potential for such bias.
(TIF)
