# Supplementary material for: Process oriented guided inquiry learning (POGIL®) marginally effects student achievement measures but substantially increases the odds of passing a course
Source: PLoS One. 2017 Oct 12;12(10):e0186203. doi: 10.1371/journal.pone.0186203 (PMC5638339; doi:10.1371/journal.pone.0186203)
Supplement: S2 Text — (DOCX) [file pone.0186203.s002.docx]

| **Author (Ref.)** | **Study Setting** | **Discipline** | **Class Size** | | | **Instructor Training** | **Included in Freeman**  **Meta-Analysis? (Ref 13)** |
| --- | --- | --- | --- | --- | --- | --- | --- |
|  |  |  | **Treatment** | **Control** | **Size** |  |  |
| Barthlow & Watson (23) | High School | Chemistry | 149 | 169 | Large | x | x |
| Brown (30) | College | Pharmacology | 78 | 66 | Medium | x | x |
| Brown et al (32) | College | Pharmacology | 14 | 59 | Small | x | x |
| Brown et al (32b) | College | Pharmacology | 23 | 59 | Small | x | x |
| Brown PJP (31) | College | Physiology | 31 | 25 | Small | x | x |
| Chase et al (22) | College | Chemistry | 93 | 178 | Medium | √ | x |
| Chase et al (22b) | College | Chemistry | 100 | 82 | Medium | √ | x |
| Eaton (21) | College | Chemistry | 47 | 33 | Small | x | x |
| Farrell et al (9) | College | Chemistry | 438 | 420 | Large | √ | √ |
| Hein (18) | College | Chemistry | 103 | 158 | Large | x | x |
| Jin & Bierm (26) | College | Non-Majors | 301 | 197 | Large | x | x |
| Kim (33) | High School | Chemistry | 25 | 26 | Small | x | x |
| Mohamed (19) | College | Chemistry | 21 | 21 | Small | x | √ |
| Murphy et al (24) | College | Chemistry | 95 | 79 | Medium | x | x |
| Murphy et al (24b) | College | Chemistry | 106 | 82 | Large | x | x |
| Perry & Wight (28) | College | Chemistry | 35 | 35 | Small | x | x |
| Pierce & Fox (17) | College | Pharmacology | 71 | 70 | Medium | x | x |
| Roller & Zori (29) | College | Nursing | 63 | 75 | Medium | x | x |
| Roller (28) | College | Nursing | 25 | 25 | Small | x | x |
| Shatila (20) | College | Chemistry | 26 | 26 | Small | x | x |
| Straumanis & Simons (10) | College | Chemistry | 96 | 739 | Medium | √ | √ |
| Straumanis & Simons (10b) | College | Chemistry | 91 | 388 | Medium | √ | x |
| Vacek (27) | College | Avaition | 19 | 11 | Small | x | x |
| Vacek (27b) | College | Avaition | 19 | 11 | Small | x | x |
| Vanags et al (25) | College | Physiology | 81 | 93 | Medium | x | x |
| Warfa & Schneider (35) | College | Chemistry | 558 | 2142 | Large | √ | x |
| Warfa & Schneider (35b) | College | Chemistry | 332 | 458 |  | √ | x |
