# Supplementary material for: Process oriented guided inquiry learning (POGIL®) marginally effects student achievement measures but substantially increases the odds of passing a course
Source: PLoS One. 2017 Oct 12;12(10):e0186203. doi: 10.1371/journal.pone.0186203 (PMC5638339; doi:10.1371/journal.pone.0186203)
Supplement: S1 Table — For studies that reported multiple outcomes, their individual effect sizes were aggregated into one summary effect size when we found the outcomes to be statistically equivalent. (DOCX) [file pone.0186203.s003.docx]

**S1 Table.** Before and after Aggregated within-study achievement effect sizes for all analyzed studies

| **Author (Ref.)** | **Sample Size (N)** | | **Individual Computed Effect Size** | | **Aggregated Effect Size** | | | **Moderators** | |
| --- | --- | --- | --- | --- | --- | --- | --- | --- | --- |
|  |  |  |  |  |  | **95% Conf. Interval** | |  |  |
|  | Treatment | **Control** | ***Hedge's g*** | **SE_g_** | ***Hedge's g*** | **Lower Limit** | **Upper Limit** | **Size** | **Domain** |
| Barthlow & Watson (23) | 149 | 169 | 0.799 | 0.014 | 0.80 | 0.57 | 1.03 | Large | Chemistry |
| Brown (30) | 73 | 66 | 0.409 | 0.029 | 0.43 | 0.10 | 0.76 | Medium | Other |
|  | 78 | 66 | 0.451 | 0.028 |  |  |  | Medium | Other |
| Brown et al (32a) | 14 | 59 | 0.136 | 0.087 | 0.28 | -0.30 | 0.86 | Small | Other |
|  | 14 | 59 | 0.429 | 0.088 |  |  |  | Small | Other |
| Brown et al (32b) | 23 | 29 | -0.007 | 0.076 | 0.45 | -0.10 | 1.00 | Small | Other |
|  | 23 | 29 | 0.904 | 0.083 |  |  |  | Small | Other |
| Brown PJP (31) | 18 | 25 | 0.039 | 0.092 | 0.58 | -0.01 | 1.17 | Small | Other |
|  | 31 | 25 | 0.760 | 0.075 |  |  |  | Small | Other |
|  | 17 | 25 | 0.948 | 0.106 |  |  |  | Small | Other |
| Chase et al (22a) | 93 | 178 | 0.275 | 0.016 | 0.06 | -0.19 | 0.31 | Medium | Chemistry |
|  | 93 | 178 | 0.166 | 0.016 |  |  |  | Medium | Chemistry |
|  | 93 | 178 | -0.078 | 0.016 |  |  |  | Medium | Chemistry |
|  | 93 | 178 | -0.080 | 0.016 |  |  |  | Medium | Chemistry |
|  | 93 | 178 | 0.020 | 0.016 |  |  |  | Medium | Chemistry |
| Chase et al (22b) | 100 | 82 | -0.116 | 0.022 | -0.06 | -0.35 | 0.23 | Medium | Chemistry |
|  | 100 | 82 | 0.015 | 0.022 |  |  |  | Medium | Chemistry |
|  | 100 | 82 | -0.183 | 0.022 |  |  |  | Medium | Chemistry |
|  | 100 | 82 | 0.050 | 0.022 |  |  |  | Medium | Chemistry |
| Eaton (21) | 39 | 33 | -0.319 | 0.055 | -0.18 | -0.63 | 0.27 | Small | Chemistry |
|  | 47 | 33 | -0.045 | 0.051 |  |  |  | Small | Chemistry |
| Hein (18) | 103 | 158 | 0.177 | 0.016 | 0.18 | -0.07 | 0.42 | Large | Chemistry |
| Jin & Bierm (26) | 301 | 197 | 0.154 | 0.008 | 0.19 | 0.10 | 0.29 | Large | Other |
|  | 301 | 197 | 0.260 | 0.008 |  |  |  | Large | Other |
|  | 301 | 197 | 0.164 | 0.008 |  |  |  | Large | Other |
|  | 301 | 197 | 0.279 | 0.008 |  |  |  | Large | Other |
|  | 301 | 197 | 0.113 | 0.008 |  |  |  | Large | Other |
| Kim (33) | 25 | 26 | 1.117 | 0.088 | 0.50 | -0.06 | 1.06 | Small | Chemistry |
|  | 25 | 26 | 0.000 | 0.076 |  |  |  | Small | Chemistry |
|  | 24 | 25 | 0.388 | 0.081 |  |  |  | Small | Chemistry |
| Mohamed (19) | 20 | 21 | 0.478 | 0.097 | 0.25 | -0.35 | 0.86 | Small | Chemistry |
|  | 20 | 21 | 0.579 | 0.098 |  |  |  | Small | Chemistry |
|  | 20 | 21 | -0.265 | 0.095 |  |  |  | Small | Chemistry |
|  | 20 | 21 | 0.223 | 0.094 |  |  |  | Small | Chemistry |
| Murphy et al (24a) | 84 | 79 | -0.311 | 0.025 | -0.32 | -0.63 | -0.02 | Medium | Chemistry |
|  | 95 | 79 | -0.333 | 0.023 |  |  |  | Medium | Chemistry |
| Murphy et al (24b) | 116 | 82 | 0.185 | 0.021 | -0.02 | -0.30 | 0.27 | Large | Chemistry |
|  | 112 | 82 | -0.219 | 0.021 |  |  |  | Large | Chemistry |
| Perry & Wight (28) | 35 | 35 | 0.312 | 0.057 | 0.31 | -0.16 | 0.78 | Small | Chemistry |
| Pierce & Fox (17) | 71 | 70 | 0.983 | 0.031 | 0.98 | 0.64 | 1.33 | Medium | Other |
| Roller & Zori (29) | 63 | 75 | 0.342 | 0.030 | 0.41 | 0.07 | 0.75 | Medium | Other |
|  | 63 | 75 | 0.485 | 0.030 |  |  |  | Medium | Other |
| Roller (28) | 25 | 25 | 0.615 | 0.081 | 0.34 | -0.22 | 0.89 | Small | Other |
|  | 25 | 25 | 0.055 | 0.078 |  |  |  | Small | Other |
| Shatila (20) | 26 | 26 | -0.267 | 0.075 | -0.17 | -0.71 | 0.38 | Small | Chemistry |
|  | 18 | 30 | -0.230 | 0.087 |  |  |  | Small | Chemistry |
|  | 26 | 28 | 0.000 | 0.072 |  |  |  | Small | Chemistry |
| Straumanis & Simons (10a) | 93 | 739 | 0.000 | 0.012 | 0.00 | -0.21 | 0.21 | Medium | Chemistry |
| Straumanis & Simons (10b) | 91 | 388 | 0.891 | 0.014 | 0.89 | 0.66 | 1.12 | Medium | Chemistry |
| Vacek (27a) | 19 | 11 | 0.013 | 0.136 | -0.21 | -0.96 | 0.55 | Small | Other |
|  | 19 | 11 | -0.923 | 0.150 |  |  |  | Small | Other |
|  | 19 | 11 | -1.025 | 0.153 |  |  |  | Small | Other |
|  | 19 | 11 | 1.102 | 0.156 |  |  |  | Small | Other |
| Vacek (27b) | 24 | 30 | 0.579 | 0.076 | 0.58 | 0.04 | 1.12 | Small | Other |
| Vanags et al (25) | 81 | 93 | 0.339 | 0.023 | 0.24 | -0.06 | 0.55 | Medium | Other |
|  | 75 | 85 | 0.148 | 0.025 |  |  |  | Medium | Other |
| Warfa & Schneider (35a) | 558 | 2143 | 0.194 | 0.002 | 0.19 | 0.10 | 0.29 | Large | Chemistry |
| Warfa & Schneider (35b) | 332 | 458 | 0.667 | 0.005 | 0.67 | 0.52 | 0.81 | Large | Chemistry |
