# Supplementary figures and images for: Process oriented guided inquiry learning (POGIL®) marginally effects student achievement measures but substantially increases the odds of passing a course
Source: PLoS One. 2017 Oct 12;12(10):e0186203. doi: 10.1371/journal.pone.0186203 (PMC5638339; doi:10.1371/journal.pone.0186203)

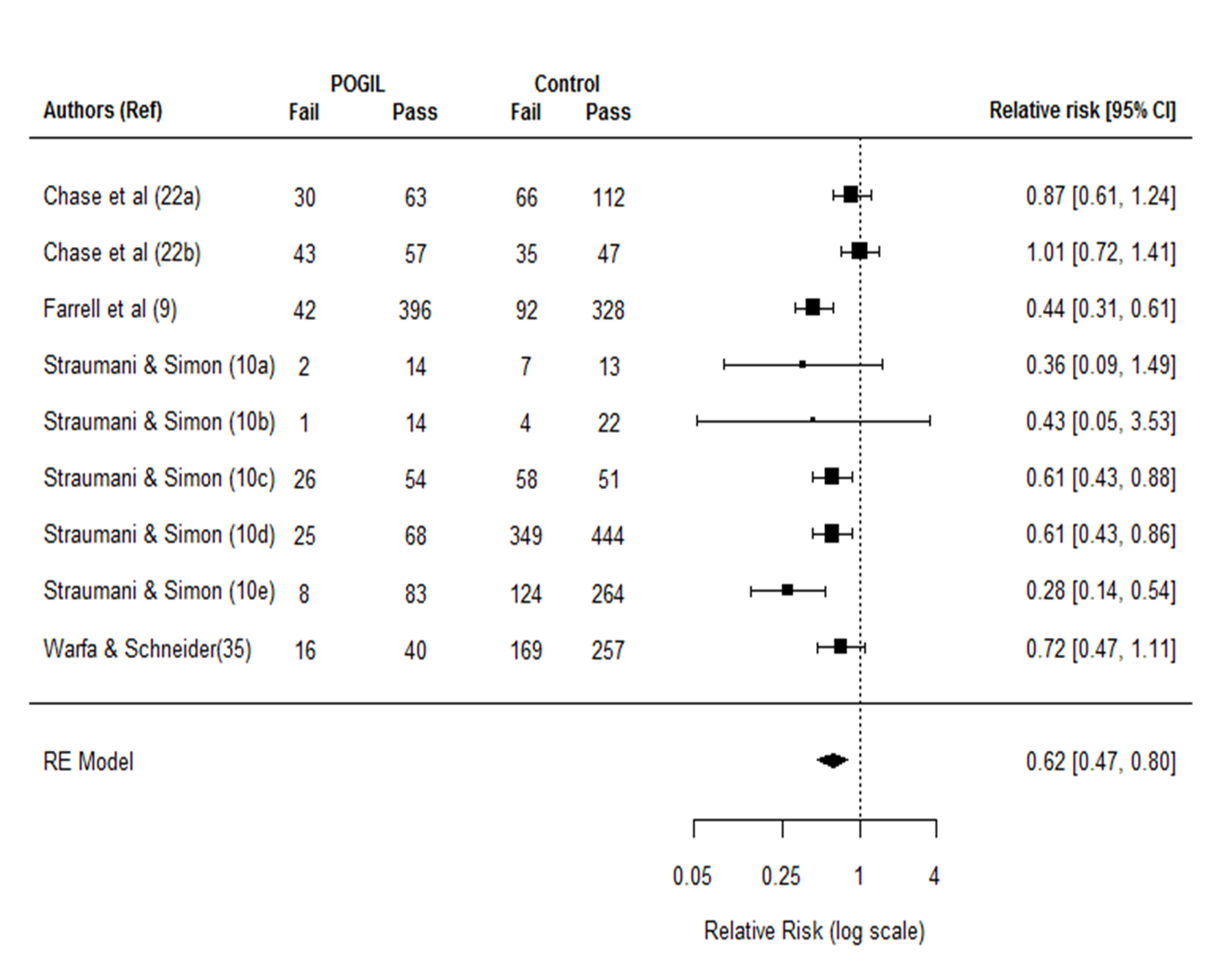

Supplement: S1 Fig — The relative risk of failing a course taught by the POGIL approach versus one taught by standard lecture is depicted as a forest plot [RR = 0.62, 95% CI: 0.47–0.80]. The POGIL approach reduced the relative risk of failure by 38%. (TIF) [file pone.0186203.s004.tif]

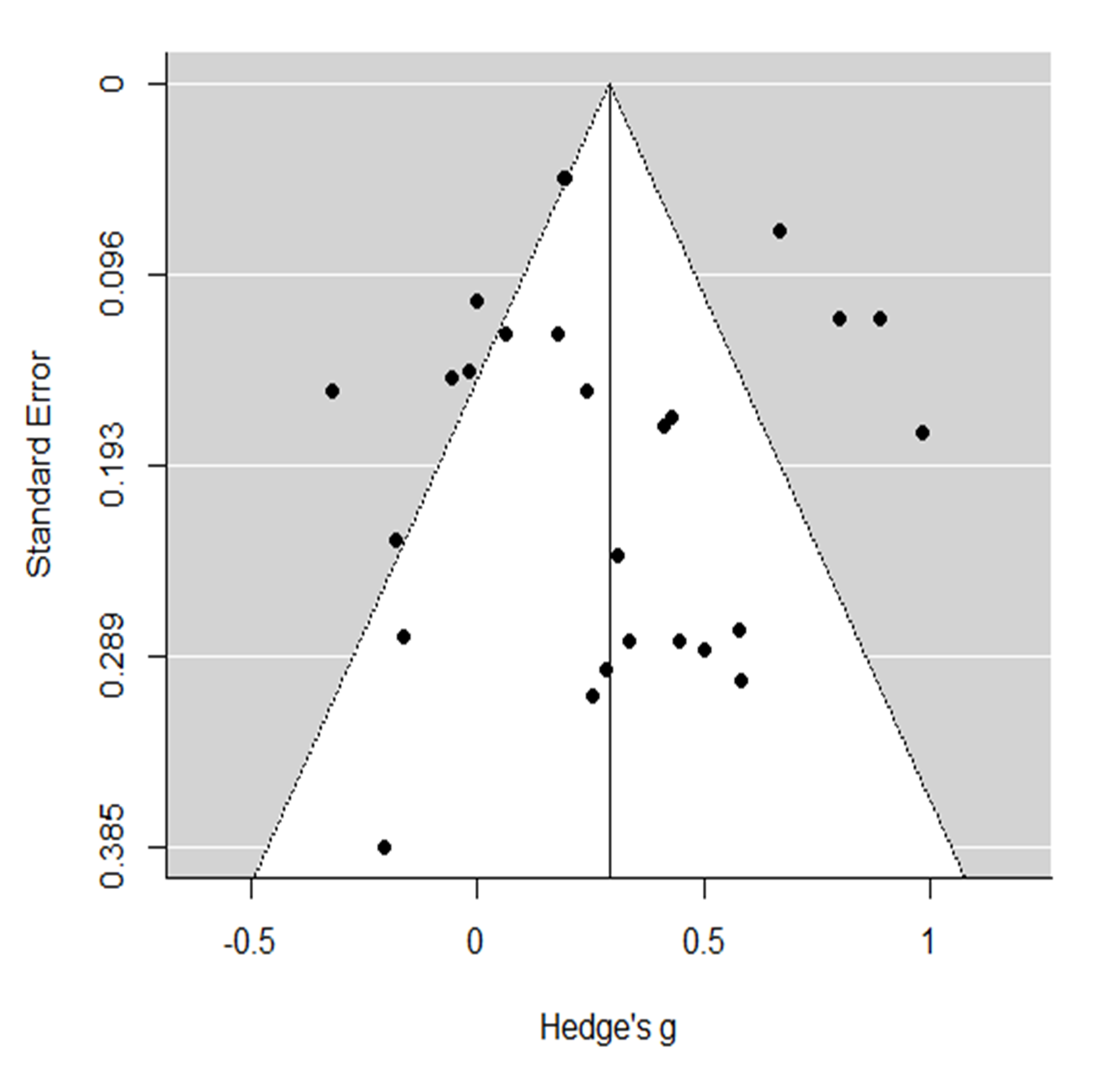

Supplement: S2 Fig — The lack of symmetry suggests the absence of publication bias even when there is potential for such bias. (TIF) [file pone.0186203.s005.tif]
